# Are There New Biomarkers in Tissue and Liquid Biopsies for the Early Detection of Non-Small Cell Lung Cancer?

**DOI:** 10.3390/jcm8030414

**Published:** 2019-03-26

**Authors:** Fiorella Calabrese, Francesca Lunardi, Federica Pezzuto, Francesco Fortarezza, Stefania Edith Vuljan, Charles Marquette, Paul Hofman

**Affiliations:** 1 Department of Cardiac, Thoracic, Vascular Sciences and Public Health, University of Padova Medical School, 35121 Padova, Italy; francesca.lunardi@unipd.it (F.L.); pezzuto.federica@libero.it (F.P.); francescofortarezza.md@gmail.com (F.F.); stefvu@hotmail.it (S.E.V.); 2 University Côte d’Azur, University Nice Hospital, FHU OncoAge, Department of Pneumology, Pasteur Hospital, 06001 Nice, France; marquette.c@chu-nice.fr; 3 University Côte d’Azur, CNRS, INSERM, IRCAN, Team 4, FHU OncoAge, 06001 Nice, France; HOFMAN.P@chu-nice.fr; 4 University Côte d’Azur, University Nice Hospital, FHU OncoAge, Laboratory of Clinical and Experimental Pathology, Pasteur Hospital, 06001 Nice, France; 5 University Côte d’Azur, Biobank (BB-0033-00025), FHU OncoAge, Pasteur Hospital, 06001 Nice, France

**Keywords:** NSCLC, biomarkers, early diagnosis, tissue, liquid biopsy

## Abstract

Lung cancer is one of the most lethal malignancies worldwide, mainly due to its late diagnoses. The detection of molecular markers on samples provided from routine bronchoscopy including several liquid-based cytology tests (e.g., bronchoaspirate, bronchoalveolar lavage) and/or on easily obtained specimens such as sputum could represent a new approach to improve the sensitivity in lung cancer diagnoses. Recently growing interest has been reported for “noninvasive” liquid biopsy as a valuable source for molecular profiling. Unfortunately, a biomarker and/or composition of biomarkers capable of detecting early-stage lung cancer has yet to be discovered even if in the last few years there has been, through the use of revolutionary new technologies, an explosion of lung cancer biomarkers. Assay sensitivity and specificity need to be improved particularly when new approaches and/or tools are used. We have focused on the most important markers detected in tissue, and on several cytological specimens and liquid biopsies overall.

## 1. Introduction

Lung cancer is the leading cause of cancer-related mortality worldwide. About 80% of lung cancer cases are non-small cell lung cancer (NSCLC), with the most frequent histotypes including adenocarcinoma and squamous cell carcinoma. Small cell carcinoma usually responds better to chemotherapy and radiotherapy, while NSCLC is commonly treated with surgery. However, the clinical outcome of conventional therapies remains very poor, largely because most patients are diagnosed with the disease either at the locally advanced or metastatic stages [1]. The five-year survival rate of advanced forms is only 4% [1]. Treatment and management of lung cancer patients constitute a serious public-health problem often with difficult-to-control health costs especially after the introduction of molecular target therapy and immunotherapy for cancer [2,3]. When diagnosed at an early operable stage, the 5-year survival rate from lung cancer climbs to above 50% [1]. 

The detection of lung cancer at early stages is therefore of great clinical value. The screening for lung cancer with chest radiography and overall low-dose computed tomography (CT) has resulted in an increased proportion of invasive procedures. Bronchoscopic examination, the principal diagnostic tool for patients with suspected carcinoma, has led to a strategy of using multiple simultaneous tests from the same bronchoscopic procedure (e.g., bronchial brushing, bronchoalveolar lavage—BAL, and endobronchial ultrasound-guided biopsy) to enhance the probability of obtaining a diagnosis. Despite this, the diagnostic accuracy of bronchoscopic examinations is sub-optimal, with the sensitivity ranging from 34–88% [4] depending on the size of the primary tumor and the number of parallel tests performed per bronchoscopy. 

Similarly, the diagnosis of lung cancer using a less invasive cytological analysis such as sputum has been reported to be prone to unsatisfactory sample collection and poor sensitivity [5]. Recently, liquid biopsy, an easily accessible minimally invasive tool, has emerged as a crucial tool in lung cancer management. 

Biomarkers detected in samples available for minimally invasive procedures and/or liquid biopsies have the potential to improve early lung cancer detection, thus allowing for early-stage cancer diagnosis and appropriate treatment. Up to 30% of surgically treated stage I patients die from recurrence [6]. The evaluation of non-invasive or tissue-based biomarkers in patients after stage I tumor resection could also identify those at a high risk of recurrence, thus, leading to improved clinical management. OMICS technologies, including genomic/epigenomics, proteomics, metabolomics and microbiomics are currently used for the more sensitive detection of novel biomarkers in many fields. Several biomarkers have recently been discovered and a growing number of other potential candidates are still awaiting the development step for better lung cancer diagnoses and management. This review mainly discusses the most relevant recently reported biomarkers that may have potential clinical applications. We have reviewed the principal biomarkers that are more consistently detected in tissue/cytological lung cancer specimens (Section 2) and liquid biopsy (Section 3), respectively. 

## 2. Potential Biomarkers in Lung Tissue/Cytological Samples 

“Biomarker” is defined as “a characteristic that is objectively measured and evaluated as an indicator of biological and pathogenic processes, or pharmacologic responses to therapeutic intervention” [7]. Advances in molecular biology and bioinformatics have resulted in the identification of a number of potential biomarkers that could be relevant in the management of patients with lung cancer. 

A number of different biomarkers have been evaluated in patients with lung cancer, including those that aimed at early disease detection or detecting minimal residual disease (diagnostic biomarkers), and those predicting both the rates of relapse and response to treatment (prognostic/predictive biomarkers) [8].

To date, all clinically approved biomarkers for lung cancer diagnoses are either proteins or peptides and all of them lack sufficient sensitivity, specificity and reproducibility even for the diagnosis of lung cancer at a late stage [9].

Similarly, the diagnosis of NSCLC using standard cytological analyses of the sputum is difficult due to the unsatisfactory sample collection and poor sensitivity [10]. Different molecular biomarkers have been tested as alternative or complementary diagnostic tools to obtain a higher sensitivity in the analysis of bronchoscopic samples and sputum. Among the wide range of molecular markers, epigenetic markers (e.g., DNA methylation, and non-coding RNA analysis) are most frequently investigated and seem to be most promising because of their crucial role in the cell cycle. 

The current article reviews publications regarding biomarkers in lung tissue/cytological samples and early lung cancer diagnoses produced in the past five years.

### 2.1. Epigenetic Biomarkers

#### DNA Methylation

Currently, the best-studied epigenetic event in the mammalian genome is DNA methylation, which is a chemical covalent modification of cytosine. In particular, it is the addition of a methyl group (CH 3) at the fifth carbon position of cytosine bases that are located 5′ to a guanosine in a CpG dinucleotide; the modification of this region interferes with gene transcription and can eliminate tumor suppressor genes. Numerous detection methods have been developed to better assay any changes. Several methylation and deacetylation inhibitors exist which can reactivate gene expression by reversing the tumor-associated epigenetic alterations [11] indicating a potential clinical use of these drugs.

Both hypomethylation and hypermethylation of well-known cancer-related genes have been found to be a process occurring in the early stages of cancer development [12,13]. 

A meta-analysis published in 2016 encompassed 151 studies on 108 genes and aimed to generate a list of differentially methylated genes between different NSCLC histotypes. The results showed two hypomethylated genes (*CDKN2A* and *MGMT*) and three hypermethylated genes (*CDH13*, *RUNX3*, *APC*) in adenocarcinomas compared with squamous cell carcinomas, with higher sensitivity and specificity values of *CDH13* and *APC* [14].

In the same year, a complementary DNA microarray analysis was performed to search for differentially expressed genes in 83 primary lung adenocarcinomas and their paired adjacent non-malignant tissues. Gene body methylation was found to be involved in the overexpression of inositol-triphosphate 3-kinase A (*ITPKA*), which regulates the inositol phosphate metabolism and calcium signaling correlated with the increased metastatic potential [15]. The authors concluded that *ITPKA* body methylation, absent in non-malignant lung tissues, appears at the premalignant stage and progressively increases with cancer development, further emphasizing its potential application for early cancer detection [15]. A panel of three tumor suppressor genes (*PCDHGB6*, *HOXA9* and *RASSF1A*) was assessed in 50 paired early-stage NSCLC by using quantum dots based on a fluorescence resonance energy transfer nanosensor technique. The combined detection was able to identify NSCLC cases with a sensitivity of 92% (in tissue samples) and 80% (in bronchial brushing), indicating the versatility of promoter expression in invasive and noninvasive NSCLC samples [16]. The *RASSF1A* methylation status was also studied in BALs obtained from 305 NSCLC patients, in combination with *SHOX2*, by using sequencing and the real-time methylation-specific polymerase chain reaction. The results of this study suggested that the methylation of these two genes has the potential to distinguish malignant lung diseases from benign and non-malignant and the combined analysis is able to increase the sensitivity of the cytological examination of lung cancer from 5.7% to 71.5% [17]. *RASSF1A* hypermethylation was also investigated in the sputum of patients with lung cancer and showed high specificity but low sensitivity [18]. The promoter methylation of another 6-cancer-specific gene panel was quantitatively investigated in sputum obtained from a prospective cohort of 210 patients (150 NSCLC patients and 60 controls), showing a high diagnostic accuracy for early-stage lung cancer, particularly for the genes *TAC1*, *HOXA17* and SOX17 [19]. Another methylation panel of six genes (SOX17, *HOXA9*, *AJAP1*, *PTGDR*, *UNCX*, *MARCH11*) was evaluated in tumor and adjacent normal lung tissue from 90 NSCLC patients showing a high sensitivity (96.7%) and specificity (60%) [20]. Interestingly, a prognostic risk category based on the methylation status of CD01, HOXAS, *PTGDR* and AJAP, refined the risk stratification for outcomes as an independent prognostic factor for early-stage diseases.

Angiotensin II type I receptor (*AGTR1*) promotor methylation was found to be another potential diagnostic biomarker. Several studies have demonstrated that *AGTR1* supports a role in regulating cell growth and proliferation during cancer development [21] in addition to its well-known effector activity in controlling blood pressure in the cardiovascular system. 

*AGTR1* was tested in both tumor (69 adenocarcinomas and 42 squamous cell carcinomas) and non-tumor tissue. Higher promoter methylation was seen in tumor tissue than in adjacent normal samples and was particularly evident in squamous cell carcinoma [22].

In a very recent study, the same group investigated a combination of target promoter sequences for the diagnosis of NSCLC by methylation-sensitive high-resolution melting analysis. In particular, 54 pairs of tumor and surrounding tissues were selected from patients with early and advanced NSCLC to determine the promoter methylation status of possible genes associated with NSCLC (*PCDHGB6*, *HOXA9*, *MGMT*, *miR-126*, *SOCS3*, *NORE1A*). The combination of *PCDHGB6*, *HOXA9*, *MGMT* and *miR-126* showed an 85.2% sensitivity and 81.5% specificity in NSCLC diagnoses, indicating that the early diagnosis of NSCLC is feasible through the monitoring of promoter methylation using an effective combination of related genes [23]. *MGMT* was also included in a literature-derived 10-methylation marker panel investigated by pyrosequencing. *L1RE1-RARB* and *L1RE1-RASSF1* (100% sensitivity and 91% specificity) were the best combinations for discriminating tumor and benign tissues when samples were of limited size such as in biopsies as well [24]. 

All DNA methylation biomarkers mentioned in this section are reported in Table 1.

### 2.2. miRNA

MiRNAs are endogenous single-stranded non-coding small RNAs with a length of ~22 nucleotides that function as antisense RNA to negatively regulate their target genes at the post-transcription level. MiRNAs are attractive as potential biomarkers as they are expressed in a tissue-specific manner and have been shown to be present in the cellular milieu and body fluids as extracellular miRNAs. The higher sensitivity and robustness against degradation by Rnase (in particular the extracellular packaging in vesicles) could promote their use as promising biomarkers for lung cancer management. Several studies have demonstrated that miRNAs are dysregulated in lung growth, recurrence and metastasis [25]. A growing number of miRNAs have been reported to be dysregulated in the early stage of cancers, sometimes before any clinical symptoms and imaging evidence [26]. These RNA molecules were originally tested particularly in the BAL, sputum [27] and tissue samples.

The first miRNA panel published in 2015 included miR-21, miR-143, miR-155, miR-210, miR372 and was evaluated in BAL obtained from NSCLC patients, showing a diagnostic sensitivity of 85.7% and specificity of 100%. Moreover, the paired comparison of sputum and BAL samples from the same patients yielded a sensitivity of 67.8% and specificity of 90%, leading to the conclusion that both tools could be used to detect early-stage NSCLC [28]. In the same period, another miRNA (miR-486) was reported to be upregulated in plasma and tissue samples obtained from patients with early-stage NSCLC [29]. Different miRNAs were evaluated in the sputum of patients that were detected by computed tomography and three of the RNA molecules (miR-21, miR-31, and miR-210) showed more than 80% sensitivity and 86% specificity in two independent cohorts [30].

One year later, two of these miRNAs (miR-21 and miR-210) and miR-372 were found to be accurate indexes for the diagnosis of patients with early or advanced NSCLC, showing a sensitivity of 67% (early forms) and 64% (advanced forms) and a specificity of 90% (early forms) and 100% (advanced forms), respectively [31]. Because of sample size limitations and variability in expression values, the authors were unable to demonstrate any meaningful association between miRNAs and the tumor subtype or patient factors.

Two important studies of the same group focused on the differential expression profiles of miRNAs in lung tumor tissue and adjacent non-tumor tissue in a large cohort of patients with lung cancer, identifying signatures for the screening of a high-risk population and early diagnoses of lung cancer. In particular, the most crucial miRNAs in cancer development were miR-205 and miR-3917 (upregulated) and miR-27a-5p, miR-30a-3p, miR-30a-5p, miR-30c-2-3p, and miR-30d-5 (downregulated) [32]. The same group identified aberrantly expressed key miRNAs in lung adenocarcinoma patients. They investigated the miR-30a-3p function more in-depth showing that it was related to in vitro cell proliferation, and thus concluded that it could be considered a promising biomarker [33]. 

More recently, exosomal miR-7, miR-21, miR-126, Let-7a, miR-17 and miR-19 obtained from the BAL fluid of patients with lung adenocarcinoma and controls have also been evaluated. The authors found an upregulation of miR-126 and Let-7a. However, only miR-126 was validated in paired tumoral and normal tissues [34]. In the same year, an interesting study appeared which investigated precursor and mature forms of the miR-944 expression in 58 tissues of NSCLC and adjacent non-cancerous specimens. A significantly higher miR-944 expression was found in NSCLC tissues, more evident in patients with squamous cell carcinoma than adenocarcinoma, suggesting that this miRNA may be involved in the early squamous-type differentiation of lung tumors and represents an accurate marker in NSCLC subtype differential diagnoses [35]. Based on the available literature, Bagheri et al. selected a panel of 11 significant noncoding RNAs to analyze their potential diagnostic role in the sputum. A logistic regression model with the best prediction was built based on miR-7, miR-126 and miR145, obtaining a 90% sensitivity and specificity in distinguishing NSCLC patients from the controls [36]. 

Table 2 reports all miRNA biomarkers mentioned in this section.

### 2.3. Proteomics

Proteomics involves the separation, identification and quantification of proteins. It also includes the analysis of proteoforms that arise as a result of post-translational modifications and sequence variants such as mutants and alternatively spliced isoforms. 

Recent advances in mass-spectrometry in combination with a range of separation methods have allowed researchers to reach satisfactory results on the different protein expressions between normal and neoplastic lung tissue [37], thus offering useful information for molecular interactions, signal pathways and biomarker identification (both diagnostic and predictive). Nevertheless, proteome complexity remains challenging. Protein dynamism (e.g., post-transcriptional modifications, alternative splicing, degradation) and the difficulties in reproducibility and repeatability represent major concerns. 

To assess the proteome status of lung cancer, several works have been carried out on BAL fluids and tissue samples. Uribarri et al. [38] identified, by using two-dimensional gel electrophoresis and subsequent protein identification by mass spectrometry (MS), a panel of 5 proteins (APOA1: apolipoprotein A-1, CO4A: complement factor 4, CRP: C-reactive protein, GSTP1: glutathione s-transferase pi 1, SAMP: serum amyloid p-component) that could accurately diagnose lung cancer on BAL, two of which interestingly could differentiate NSCLC and SCLC. A larger panel was investigated a year later by Almatroodi et al. [39]. Using liquid chromatography-mass spectrometry (LC)-MS, these authors also detected an up-regulation of 33 proteins in all tumor samples from adenocarcinoma compared to the controls, suggesting a certain role as a potential biomarker. Focusing on the earlier steps of carcinogenesis, Kato et al. compared the proteomic profiles obtained from tissues of three subtypes of lepidic-type adenocarcinoma (in situ, minimally invasive and lepidic-predominant invasive). The distinct protein profile differentially expressed in each histotype (70 proteins for LPIA including HPX, CTTN, CDH1, EGFR, MUC1, 15 proteins for MIA including CRABP2, LMO7, RNPEP and 26 proteins for AIS including LTA4H and SOD2) led to a deeper awareness of the lepidic-predominant invasive tumor as separate from pre-invasive lesions and to a better knowledgeableness of which proteins are involved in early-stage progression [40]. 

A combination of a quantitative proteomic and transcriptomic analysis was carried out by Tenzer et al. on tumor samples of adenocarcinoma, squamous cell carcinoma and control cases [41]. The authors identified 12 proteins specifically expressed in tumor samples and, similarly, three proteins that were higher in all controls. In addition, squamous cell carcinoma and adenocarcinoma have demonstrated a characteristic protein signature that could differentiate the two subtypes.

Hsu et al. identified 133 protein candidates from paired adenocarcinoma tissues with different extents of lymph node involvement by iTRAQ (isobaric tags for relative and absolute quantification)-labeling technology coupled with 2D-LC-MS/MS (two-dimensional LC-tandem MS) [42]. They further validated six potential biomarkers (ERO1L: endoplasmic reticulum oxidoreductin 1, NARS, PABPC4: poly(A)-binding proteins, RCC1: regulator of chromosome condensation 1, RPS25: ribosomal protein S25, and TARS: threonyl-TRNA synthetase) which were highly expressed in adenocarcinoma tissues compared to adjacent normal tissues. In addition, they found ERO1L and NARS to be positively associated with lymph node metastasis, in which ERO1L overexpression in patients with early-stage adenocarcinoma was associated with poor overall survival.

Ortea et al. tested a novel quantitative proteomics approach on BAL [43]. They detected a panel of 44 proteins differentially expressed in adenocarcinoma and in the control group, among which there were HPT (haptoglobin), CO4A and GTSP1, consistent with what had already been reported before. 

More recently, Carvalho et al. [44] performed LC-MS to demonstrate 133 proteins that were significantly different between neoplastic and non-neoplastic BAL and an overlap with biomarkers detected in lung tissue.

Proteomic analysis with LC-MS and validation with PMR-MS (parallel reaction monitoring mass spectrometry) and immunohistochemistry in a tumor microarray was used by Codreanu et al. [45] to compare adenocarcinoma samples and benign nodules. Seven proteins (ALOX5: arachidonate 5-lipoxygenase, ALOX5AP: arachidonate 5-lipoxygenase-activating protein ITGAX: integrin alpha X, SLC2A3: solute carrier family 2, CEACAM6: carcinoembryonic antigen-related cell adhesion molecule 6, CRABP2: cellular retinoic acid-binding protein 2, LAD1: leukocyte adhesion deficiency-1) were identified as possible key discriminant biomarkers among the groups. 

Protein involvement in premalignant lesions has been further investigated by Nan et al. [46]. The authors identified ten proteins specifically in squamous metaplasia and atypical adenomatous hyperplasia. The overexpression of FAK (focal adhesion kinase) and C-src, two tyrosine-protein kinases, were reported as potentially detectable for early diagnoses of lung cancer. 

All proteomic biomarkers mentioned in this section are reported in Table 3.

### 2.4. Metabolomics 

In the post-genomic era, metabolomics is a new technology developed to determine small molecule intermediates and products of metabolism in organisms. The complete set of metabolites, which are defined as the end products of cellular processes, represents the metabolome. In contrast to mRNA gene expression data and proteomics, metabolomics faithfully and accurately reflects the cellular physiology. Several efforts have been made concerning the application of metabolomics in cancer research to identify novel metabolites useful as biomarkers. It appears relevant especially for cancers that are difficult to diagnose in the early stage, such as lung cancer. Two main techniques are usually used to analyze metabolites in lung cancer, singularly or in combination: mass spectrometry and nuclear magnetic resonance. Recently, the development of a next-generation metabolomic technique has improved the sensitivity and accuracy of the method. Focusing on the detection of early-stage lung cancer, the markers should be able to distinguish between neoplastic and healthy patients with high sensitivity and specificity.

The detection of metabolites has been tested both on cytological specimens and lung tissues. 

Changes in metabolite characteristics have been shown by Li et al. The authors developed an in situ metabolomics method based on MS imaging by using air flow-assisted desorption electrospray ionization (AFADESI) through which they discriminated choline and carnitine as endogenous metabolites differentially expressed between cancerous and normal adjacent lung tissue, as well as in terms of spatial distribution [47]. Focusing on adenocarcinoma, Wikoff et al. revealed a perturbation in the metabolism of glucose, cysteine and glucosamine associated with early-stage lung cancer compared to non-malignant tissues [48]. From a comparison with benign pulmonary tissues performed by HR ^1^H NMR spectroscopy, Chen et al. identified a significant increase in the lipids and lactate levels and a decrease of inositol and valine in the cancerous tissue areas [49]. Several metabolites have emerged as potential diagnostic markers detected by a metabolomic approach with two mass-spectrometry platforms (gas chromatography (GC-MS)/MS and flow injection electrospray (FIE)-MS) on spontaneous sputum [50]. Clinical samples and controls differed for polyamine metabolites (e.g., putrescine), lipid metabolites (e.g., glycerophospholipids), isobutyl decanoate and diethyl glutarate. Moreover, the levels of hexanal, cysteic acid, hydroxypyruvic acid and cholesterol ester with an acyl group CE were different between lung cancer samples and non-neoplastic diseases. Interestingly, ganglioside GM1, a glycosphingolipid previously linked to lung cancer, reached the highest value of diagnostic accuracy. Based on these results the authors proposed that metabolic activity of BAL provides more information on epithelial surfaces of the lower respiratory tract. A comparison of the metabolic pathways in lung cancer and control BALs by DI-ESI-QTOF-MS (direct infusion high-resolution MS) and GC-MS identified 42 metabolites in neoplastic samples, with mainly an alteration in glutamate/glutamine metabolism [51]. A further investigation selected phosphoric acid and glycerol as suitable sensitive and specific molecular biomarkers.

More recently, broad research on lung tissue has been performed by Moreno et al. to find the metabolic differences between neoplastic (adenocarcinoma and squamous cell carcinoma) and normal tissue. Several biochemical pathways and metabolites, such as nucleotides, showed significant changes with high predictive capability values, mainly concerning the distinction between tumor versus nonmalignant tissue and also the intrinsic changes in lung cancer subtypes [52]. 

Table 4 reports all metabolomic biomarkers mentioned throughout this section.

### 2.5. Microbiome

The number of bacteria in the body is estimated to be of the same order as the number of human cells [53] and the combined genetic material of the microorganisms is defined as the microbiome. Advances in genome sequencing and metagenomic analysis have enabled researchers to study the microbiome and its relationship with the human environment. Alteration of the host-bacterial symbiosis is related to several systemic diseases (inflammatory bowel diseases, diabetes, etc.) and it has been found to be involved in carcinogenesis, interacting in metabolic, inflammatory or immune pathways. The link between the microbiome and cancer has been demonstrated by several studies, especially in gastrointestinal malignancies [54].

Much evidence confirms the role of the microbiome profile in the development of lung cancer, suggesting its possible use as a biomarker in tissue or cytological samples for early detection. 

In 2016, Yu et al. characterized the taxonomic profiles of lung microbiome in non-malignant and malignant lung tissue samples from a cohort of lung cancer patients. The authors found that the alpha diversity (number of different species) was significantly higher in non-neoplastic than in neoplastic tissue. Moreover, the microbiome composition could be correlated with the histotype and tumoral progression. In adenocarcinoma, an abundance of *Thermus* and decreased levels of *Ralstonia* compared to squamous cell carcinoma was observed. In the same study, the authors found that the levels of *Legionella* were high in metastatic cases, hypothesizing a possible role in metastatic spread [55].

Similar data were obtained by an independent research group. They studied a smaller group and found that the genus *Streptococcus* was significantly more abundant in cancer samples than in the controls, while *Staphylococcus* was more represented in the control group [56].

Alteration of the microbiome can be caused by a somatic genetic mutation induced by smoking. In a recent work, Greathouse et al. studied the interaction between the microbiome and TP53 in a smoker with lung cancer. The authors found an abundance of *Acidovarax* in squamous cell carcinoma tissue with a TP53 mutation, an association not seen in adenocarcinoma [57].

The microbiome was also investigated in salivary samples. Significantly altered levels of *Capnocytophaga*, *Selenomonas*, *Veillonella* and *Neisseria*—commensal species of the upper digestive and respiratory tracts—were found in patients with lung adenocarcinoma and squamous cell carcinoma compared to a control group. Interestingly, the combined high levels of *Capnocytophaga* and *Veillonella* in the saliva were strongly associated with lung cancer, suggesting a role as a biomarker for early detection [58].

The metagenomic sequencing of the sputum microbiome identified higher levels of *Streptococcus viridans* and another 16 species in lung cancer samples. Particularly, *Granulicatella adiacens*, a commensal bacterium and an opportunistic human pathogen, was more represented [59].

Only one work has focused on the microbiome in the BAL fluid of lung cancer patients. The authors highlighted a different composition of bacterial commensal communities, detecting two phyla, *Firmicutes* and *TM7*, and two genera, *Veillonella* and *Megasphaera*, in the BAL of lung cancer patients compared to the samples of patients with benign masses. Increased phylum TM7 was also observed in both chronic obstructive pulmonary disease (COPD) and lung cancer patients, suggesting a potential role for TM7 in the transformation of COPD to lung cancer [60].

Microbiome biomarkers described in this section are reported in Table 5.

### 2.6. What is the Future for Tissue/Cytological Biomarkers for Detecting Lung Cancer at the Early Stage?

A broad armamentarium of highly sensitive technologies has been used to discover different molecular biomarkers for earlier diagnoses of NSCLC. However, despite the number of relevant studies in the epigenetic field, the exact potential impact of DNA methylation and miRNA as biomarkers still remains difficult to accurately estimate: meta-analyses are difficult due to the large diversity of samples, detection methods, and inclusion criteria. 

Proteomic and metabolomic approaches have the potential to be used as valid molecular diagnostic tools for early lung cancer detection although more effort should be employed to standardize the processing and analysis of large numbers of data. 

Microbiome in lung cancer is still in its infancy. Most of the described studies were conducted on small cohorts.

Many of these potential biomarkers, especially the new ones, need to be further studied to enter the trial area or clinical practice. In the future, more effort should be made to favor multicenter studies for validation.

The role of these tissue/cytological biomarkers in the early diagnoses of lung cancer comes up against the “barrier” of the sampling technique. For instance, in contrast with low dose computed tomography (LDCT), which clearly showed a clear cut benefit in terms of the survival in the setting of lung cancer screening, autofluorescence bronchoscopy, when used in combination with LDCT to enhance the detection rate of central airway cancers, has finally failed to yield any benefit in high lung cancer-risk patients screening [61].

## 3. Potential Biomarkers from Liquid Biopsy

Liquid biopsy (LB) is a new potential noninvasive tool for the detection of diagnostic biomarkers in patients with early-stage lung cancer or in patients with a high risk of lung cancer onset. In this context, different blood compartments and components have been explored in many studies, mostly separately [62,63,64]. Thus, recent publications and reviews highlight that the detection and/or the characterization of circulating tumor cells (CTCs), circulating miRNAs, exosomes, circulating free DNA, platelets and/or proteins in plasma, may participate in the detection of early-stage lung cancer [62,63,64,65]. These circulating biomarkers demonstrate a strong interest in two domains. First, they could have an added value in the diagnosis of lung cancer, notably when one or several nodules of uncertain malignancy are observed on a chest CT scan. Therefore, without a histological/cytological diagnosis of lung cancer, a biological signature may help in the surgical decision to remove a lung with a suspicious nodule in association with radiological criteria [62,63]. Additionally, a biological signature in blood might help in the prediction of lung cancer onset in higher risk populations, such as heavy smokers, patients with COPD and patients over 55 years of age [63].

In the following section, the most important articles about biomarkers in LB and early lung cancer diagnoses have been reported (Table 6).

### 3.1. Circulating Tumor Cells

Circulating non-hematological cells (CNHC), most often called “circulating tumor cells” (CTCs), can be isolated in patients with early-stage lung carcinoma [66,81]. The detection and characterization can be made by different methods such as the cytomorphological and phenotypic assessment of the different CNHC populations after blood filtration [81]. Previous studies by our team showed that CTCs could be detected before the surgical resection of lung cancer in more than 50% of cases using blood filtration through polycarbonate filters [66,67]. Other investigators also demonstrated using different techniques that CTCs can be isolated in patients with early-stage lung carcinoma [68]. It should be highlighted that according to the method used, the percentage of detected CTCs can often be variable [67]. As an example the number of CTCs detected in lung cancer patients is much lower when using the CellSearch approach than when using blood filtration through polycarbonate filters [67]. This is due to the fact that the CellSearch method is based on the capture of CTCs expressing cytokeratins by using ferrofluids associated with the Epcam antibody. Since a number of these CTCs undergo an epithelial to the mesenchymal phenomenon, the CellSearch method can miss the detection of a couple of CTCs [67].

One previous pilot study showed that CTCs could be detected in COPD patients without any detectable lung nodule on a chest CT scan [69]. In this study, the cytological assessment of CTCs observed on the surface of polycarbonate filters was based on the cytological criteria of malignancy [69]. These latter detected cells were called “sentinel cells” since they were isolated in the blood of patients several weeks before the onset of a nodule at the chest CT scan. A French national multicenter study is currently ongoing for the validation of these initial results [82].

### 3.2. Plasma miRNA 

Several studies have demonstrated that some more or less complex signatures of plasma miRNA were associated with the presence of lung carcinoma or could discern the malignancy of a lung nodule with an uncertain diagnosis during CT scan examination [70,71,83,84]. Shen et al. demonstrated that miR-21 and miR-210 were upregulated and that miR486-5p was downregulated in malignant solitary pulmonary nodules compared with benign solitary pulmonary nodes [70]. A model with a 75% sensitivity and 85% specificity for detecting malignancy was then proposed by these authors [83]. Interestingly, miRNA signatures from the blood were found to be specific for lung cancer but also for a high risk of developing lung carcinoma [72,73]. In one study, Boeri et al. identified that a panel of 15 miRNAs in blood for detecting lung carcinoma prior to diagnosis and another panel of 13 miRNAs for lung cancer diagnoses [72]. It is noteworthy that usually there is not a higher concordance between the miRNAs overexpressed or underexpressed in the plasma samples and their corresponding expression in the tumor tissue samples. However, some publications demonstrated that some detectable miRNAs in blood samples (such as miR17-3p, miR21, miR106a, miR146, miR155, miR199, miR203, miR192, miR205, miR210, miR212, and miR214) were present at a high level in lung tumor tissues too [84].

### 3.3. Circulating Free DNA

Different studies have shown a strong link between some genomic alterations detected from circulating free DNA and the presence of early-stage lung carcinoma [74,83,85]. Methylation analysis in the plasma DNA of certain genes (*SOX17*, *TAC1*, *HOXA7*, *SOX17*, *HOXA9 ZFP42h*) using quantitative methylation-specific real-time PCR and methylation-on-beads for cancer-specific genes showed a strong association with the diagnosis of early-stage lung carcinoma [19]. In this study, the authors detected a promoter methylation sensitivity and specificity of 93% and 62%, respectively, using plasma [19].

### 3.4. Other Circulating Biomarkers of Potential Interest

Several studies have looked for blood antibodies associated with the presence of lung carcinoma [70,71,72,73,74,75,76,83,84,85]. One of these studies identified five epitopes from a phage display library of lung carcinomas that strongly reacted with the plasma from patients with NSCLC compared with healthy individuals [76]. Additionally, some plasma protein signatures have been characterized as predictive biomarkers of lung cancer onset or have been associated with early-stage lung carcinoma [77,83]. Exosomes which are cell-derived vesicles with diameters of 30–100 nm can be detected in various body fluids including plasma [86,87]. These circulating particles are still a new field of study, and no well-established protocols are currently available for their isolation and characterization. However, exosomes will certainly play a role not only in the diagnosis and prognosis of lung cancer but also in the screening of this cancer [78,79,80]. Finally, other components of the blood such as platelets can be potentially useful for the detection of early stages of lung cancer [88]. 

### 3.5. What Is the Future for the Use of Liquid Biopsies to Detect Lung Cancer at an Early Stage? 

Currently, there is no biological test of the blood that has been validated in routine clinical practice for the detection of early-stage lung carcinoma. In this context, the number of circulating biomarkers that can be detected in blood shows the complexity of this field, particularly for applications in the health care field. One major challenge is to combine different biomarkers from different components at the same time for one patient. This issue also needs to master the pre-analytical steps before the analyses [89]. Finally, the integration of these combined and complex analyses will most probably be supported in the near future by in-depth education and machine learning approaches based on artificial intelligence [90].

## 4. Concluding Remarks

Much effort has been made in recent years to identify biomarkers for the early diagnosis of NSCLC in order to improve the clinical management and survival of cancer patients. However, some pitfalls concerning the design and overall validation of biomarkers have certainly delayed their transfer to the clinical setting. 

A broad armamentarium of new Omic technologies has been used in different types of specimens (lung tissue, cytological samples, liquid biopsy) often lacking standardization and including a small cohort of patients.

In conclusion, better knowledge and standardization of new technologies on large case series are mandatory for the right selection of biomarkers.

## Figures and Tables

**Table 1:** The DNA methylation biomarkers for the “early” diagnosis of lung cancer.

Authors	PubMed ID	Markers	Source and Type of Tumor	AUC	Sensitivity	Specificity
Huang T [14]	26862903	*CDH13* *APC* *CDKN2A* *MGMT* *RUNX3*	- Meta-analysis of 151 studies on 108 genes - Tissues - AC vs. SSC	0.68 (*CDH13*) 0.66 (*APC*) 0.45 (*CDKN2A*) 0.40 (*MGMT)* 0.47 (*RUNX3*)	0.49 0.60 0.37 0.32 0.47	0.74 0.65 0.55 0.60 0.86
Wang YW [15]	27234602	*ITPKA*	- Tissues - 83 AC vs. adjacent normal tissues - Cell lines (NSCLC, SCLC)	n.a.	n.a.	n.a.
Ma Y [16]	27240011	*PCDHGB6*, *HOXA9*, *RASSF1A*	- Tissues (50 NSCLC vs. adjacent normal tissues) - Bronchial brushing (NSCLC vs. healthy patients)	0.977 * 0.907 **	0.92 0.80	1.0 1.0
Ren M [17]	28325362	*RASSF1A* *SHOX2*	- BAL - 305 patients with lung cancer, benign lung lesions, other solid organ cancer or no exact diagnosis	n.a.	0.50 (*RASSF1A*) 0.64 (*SHOX2*)	0.96 0.92
Hubers AJ [18]	27496969	*RASSF1A* 3OST2 *PRDM14*	- Sputum - 1548 patients screened for lung cancer development within 2 years	n.a.	0.28	0.90
Hulbert A [19]	27729459	*TAC1*, *HOXA17*, SOX17	- Sputum - 210 patients	0.89	0.98	0.71
Ooki A [20]	28855354	SOX17, *HOXA9*, *AJAP1*, *PTGDR*, *UNCX*, *MARCH11*	- Tissues - 133 NSCLC vs. adjacent normal tissues	n.a.	0.97	0.60
Chen R [22]	29085512	*AGTR1*	- Tissues - 111 NSCLC vs. adjacent normal tissue - AC vs. SCC	n.a.	n.a.	n.a.
Liu F [23]	29725463	*PCDHGB6*, *HOXA9*, *MGMT*, *miR-126*	- Tissues - 54 NSCLC vs. adjacent normal tissues - AC vs. SCC	0.89	0.85	0.81
Walter RFH [24]	29851970	*L1RE1-RARB*, *L1RE1-RASSF1*	- Tissues - 116 lung cancer vs. 22 normal tissues	n.a.	1.0	0.91

AC, adenocarcinoma; AUC, the area under the curve; BAL, bronchoalveolar lavage; n.a., not available NSCLC, non-small cell lung cancer; SCC, squamous cell carcinoma. *, tissues, **, bronchial brushing.

**Table 2:** The miRNA biomarkers for the “early” diagnosis of lung cancer.

Authors	PubMed ID	Markers	Source and Type of Tumor	AUC	Sensitivity	Specificity
Kim JO [28]	25862841	miR-21, miR-143, miR-155, miR-210, miR372	- BAL and sputum - 21 NSCLC patients vs. 10 controls	n.a.	0.86 * 0.68 **	1.0 * 0.90 **
Li W [29]	26237047	miR-486	- Plasma and tissue - 11 NSCLC patients vs. 11 controls	0.926 (miR-486) ^#^	0.91 ^#^	0.82 ^#^
Xing L [30]	25593345	miR-21, miR-31, miR-210	- Sputum - 203 NSCLC patients vs. 210 patients with benign nodule	0.919	0.83	0.88
Razzak R [31]	27122989	miR-21, miR-210, miR-372	- Sputum - 21 early and 22 advanced NSCLC patients vs. 10 controls	n.a.	0.67 ^◦^ 0.64 ^◦◦^	0.90 ^◦^ 1.0 ^◦◦^
Zhang Y [32]	28498428	miR-205, miR-3917, miR-27a-5p, miR-30a-3p, miR-30a-5p, miR-30c-2-3p, miR-30d-5	- Tissue - 81 NSCLC vs. adjacent normal tissues	0.919	n.a.	n.a.
Sui J [33]	28791371	miR-30a-3p, miR-96-5p, miR-182-5p, miR-30c-2-3p, miR-221-5p	- Tissue - 53 AC vs. adjacent normal tissues	0.837 (miR-30a-3p) 0.819 (miR-96-5p) 0.835 (miR-182-5p) 0.674 (miR-30c-2-3p) 0.546 (miR-221-5p)	n.a.	n.a.
Kim JE [34]	29806739	miR-7, miR-17, miR-19, miR-21, miR-126, Let-7a	- Exosomes from BAL - 4 Tissue samples - 13 AC vs. 15 controls	n.a.	n.a.	n.a.
Powrozek T [35]	29496309	Pri-miR-944 miR-944	- Tissue - 58 NSCLC vs. adjacent normal tissues - SCC vs. AC; SCC vs. normal	0.978 ^◊^ 0.992 ^◊◊^	0.93 ^◊^ 0.93 ^◊◊^	1.0 ^◊^ 1.0 ^◊◊^
Bagheri A [36]	30485511	miR-7, miR-126, miR-145	- Sputum - 30 NSCLC patients vs. 30 controls - SCC vs. AC	0.93	0.9	0.9

AC, adenocarcinoma; AUC, the area under the curve; BAL, bronchoalveolar lavage; n.a., not available NSCLC, non-small cell lung cancer; SCC, squamous cell carcinoma. *, BAL, **, sputum; ^#^, plasma; ^◦^, early NSCLC, ^◦◦^, advanced NSCLC; ^◊^, SCC vs. AC, ^◊◊^, SCC vs. normal.

**Table 3:** The proteomic biomarkers for the “early” diagnosis of lung cancer.

Authors	PubMed ID	Markers	Source and Type of Tumor	AUC	Sensitivity	Specificity
Uribarri M [38]	25105437	APOA1, CO4A, CRP, GSTP1, SAMP	- BAL - 139 lung cancer vs. 49 controls - 43 SCLC vs. 96 NSCLC	0.94	0.95	0.81
Almatroodi SA [39]	25560643	33 proteins	- BAL - 8 AC vs. 8 controls	n.a.	n.a	n.a
Kato Y [40]	26162278	70 proteins for LPIA 15 proteins for MIA 26 proteins for AIS	- Tissue - 3 AIS vs. 3 MIA vs. 3 LPIA	n.a	n.a	n.a
Tenzer S [41]	26930711	12 proteins in tumor tissue; 3 proteins in controls	- Tissue - 21 NSCLC vs. adjacent normal tissue - 11 AC vs. 10 SCC	0.92–0.00	n.a	n.a
Hsu CH [42]	27161446	EROL1, PABPC4, RPS25, TARS, NARS, RCC1	- Tissue - 14 AC vs. adjacent normal tissue	n.a	n.a	n.a
Ortea I [43]	26917472	44 proteins	- BAL - 12 AC vs. 10 controls	0.917–0.525	n.a	n.a
Carvalho AS [44]	28169345	133 proteins	- BAL - 90 suspected lung cancer prospectivelyt followed for two years	n.a	n.a	n.a
Codreanu SG [45]	28731711	7 proteins	- Tissue - 34 benign nodules vs. 24 AC, vs. 5 normal bronchial vs. 5 normal alveolar epithelium	0.96–0.61	n.a	n.a
Nan Y [46]	26809240	10 proteins	- Tissue - 6 AC vs. 7 SCC	n.a	n.a	n.a

AC, adenocarcinoma; AUC, the area under the curve; BAL, bronchoalveolar lavage; n.a., not available; NSCLC, non-small cell lung cancer; SCC, squamous cell carcinoma; LPIA, lepidic predominant invasive adenocarcinoma; MIA, minimally invasive adenocarcinoma; AIS, adenocarcinoma in situ; SCC, squamous cell carcinoma.

**Table 4:** The metabolomic biomarkers for the “early” diagnosis of lung cancer.

Authors	PubMed ID	Markers	Source and Type of Tumor	AUC	Sensitivity	Specificity
Li T [47]	26404114	choline, [PC(38:2) + Na]^+^, [PC(16:0/20:4) + Na]^+^, and [PC(36:3) + Na]^+^, [PC(34:1) + K]^+^, [PC(18:1/20:3) + Na]^+^	- Tissues - 52 NSCLC vs. 21 adjacent normal tissues - 37 AC vs. 15 SCC	0.968	0.94	1.0
Wikoff WR [48]	25657018	20 annotated and 22 structurally unknown metabolites	- Tissues - 39 early stage AC vs. adjacent normal tissues	0.885	0.923	0.846
Chen W [49]	27002768	Lactate, lipids, myo-inositol and valine	- Tissues - 14 AC; 16 SCC; 4 others	0.90–0.60	n.a.	n.a.
Cameron SJ [50]	26973212	Hexenal, cysteic acid, hydroxypyruvic acid, cholesterol ester with an acyl group CE	- Sputum - 23 lung cancer (16 NSCLC, 6 SCLC, 1 radiological dg), vs. 11 non neoplastic but suspected lung cancer vs. 33 controls	>0.80	n.a.	n.a.
Callejon-Leblic B [51]	27255828	42 altered metabolites	- BAL - 24 lung cancer vs. 31 controls	0.87–0.50	n.a.	n.a.
Moreno P [52]	30099851	AC: 5,6 dihydrouracil, Inosine, Adenosine 5′ monophosphate, Xanthosine, 2′ deoxyinosine SCC: 2′ O methylguanosine, 5 methyluridine, 5,6 dihydrothymine, 2′ deoxyuridine, 2′ deoxyinosine	- Tissues - 68 lung cancers vs. 68 normal tissue - 33 AC vs. 35 SCC	-	0.79–0.90 0.94–1.0	0.79–0.90 0.97–1.0

AC, adenocarcinoma; AUC, the area under the curve; BAL, bronchoalveolar lavage; n.a., not available NSCLC, non-small cell lung cancer; SCC, squamous cell carcinoma; PC, phosphatidylcholine; CE, cholesteryl ester.

**Table 5:** The microbiome biomarkers for the “early” diagnosis of lung cancer.

Authors	PubMed ID	Markers	Source and Type of Tumor	AUC	Sensitivity	Specificity
Yu G [55]	27468850	*Proteobacteria*, *Thermus*, *Legionella*	- Tissues - 165 non neoplastic lung tissue from cancer patients (97 AC, 63 SCC, 5 mixed type)	-	-	-
Liu et al. [56]	29023689	*Streptococcus*	- Tissue - 19 NSCLC (12 AC, 7 SCC) vs. 5 SCLC; vs. 18 controls	0.693	87.5%	55.6%
Greathouse KL [57]	30143034	*Acidovarax*	- Tissue - 143 lung cancer vs. 33 controls 67 AC vs. 47 SCC vs. 29 other	-	-	-
Yan X [58]	26693063	*Capnocytophaga*, *Veillonella*	- Saliva - 61 NSCLC vs. 25 controls - 38 AC vs. 23 SCC	0.86 *	84.6% *	86.7% *
				0.80 **	78.6% **	80.0% **
Cameron SJS [59]	28542458	*Granulicatella adiacens*	- Sputum - 10 patient with lung cancer-like symptoms (6 LC-, 4 LC+)	-	-	-
Lee SH [60]	27987594	*Veillonella*, *Megasphaera*	- BAL - 18 NSCLC (13 AC, 5 SCC) vs. 2 SCLC vs. 8 benign mass-like lesion	0.888	70.0–95.0%	75.0–100%

* SCC from control; ** AC from control; AC, adenocarcinoma; AUC, the area under the curve; BAL, bronchoalveolar lavage; n.a., not available NSCLC, non-small cell lung cancer; SCC, squamous cell carcinoma.

**Table 6:** The biomarkers from the liquid biopsy for the “early” diagnosis of lung cancer.

Authors	PubMed ID	Markers	Study Population
Hofman V [66]	21098695	CTCs	208 NSCLC patients and 39 healthy subjects
Hofman V [67]	21128227	CTCs	210 NSCLC patients
Xue Y [68]	30589049	CTCs	72 NSCLC patients
Ilie [69]	25360587	CTCs	168 COPD patients and 77 healthy subjects
Shen J [70]	21864403	miRNAs	108 patients with malignant nodules and 113 patients with benign lung nodules
Yu H [71]	30259714	miRNAs	Meta-analysis of 17 studies
Boeri M [72]	21300873	miRNAs	Training set = 38 lung cancer patients and validation set = 53 lung cancer patients
Sozzi G [73]	24419137	miRNAs	939 participants including 69 patients with lung cancer and 870 disease-free individuals
Sozzi G [74]	18787214	Plasma DNA	1035 subjects, 956 cancer free 38 with lung cancer, and 41 with other tumors
Hulbert A [19]	27729459	Plasma DNA and Sputum	150 lung cancer patients and 60 healthy subjects
Boyle P [75]	20675559	Plasma antibodies	525 lung cancer patients
Zhong L [76]	17409910	Plasma antibodies	23 lung cancer patients and 23 healthy subjects
Guida F [77]	30003238	Plasma proteins	108 ever-smoking patients with lung cancer diagnosed within 1 year after blood collection and samples from 216 smoking-matched controls
Vykoukal J [78]	29221141	Plasma-derived extracellular vesicle proteins	13 lung adenocarcinoma and 15 controls
Cazzoli A [79]	23945385	MicroRNAs from circulating exosomes	Training set: 10 adenocarcinomas, 10 lung granulomas and 10 healthy former smokers Validation set: 50 adenocarcinomas, 30 lung granulomas, 25 healthy former smokers
JinX [80]	28606918	Exosomal miRNAs	46 stage I NSCLC patients and 42 healthy individuals

CTCs, circulating tumor cells; NSCLC, non-small cell lung cancer; miRNA, microRNA; COPD, chronic obstructive pulmonary disease.
